# The orthotopic xenotransplant of human glioblastoma successfully recapitulates glioblastoma-microenvironment interactions in a non-immunosuppressed mouse model

**DOI:** 10.1186/1471-2407-14-923

**Published:** 2014-12-08

**Authors:** Celina Garcia, Luiz Gustavo Dubois, Anna Lenice Xavier, Luiz Henrique Geraldo, Anna Carolina Carvalho da Fonseca, Ana Helena Correia, Fernanda Meirelles, Grasiella Ventura, Luciana Romão, Nathalie Henriques Silva Canedo, Jorge Marcondes de Souza, João Ricardo Lacerda de Menezes, Vivaldo Moura-Neto, Fernanda Tovar-Moll, Flavia Regina Souza Lima

**Affiliations:** Instituto de Ciências Biomédicas, CCS – Bloco F, Universidade Federal do Rio de Janeiro, 21949-590 Rio de Janeiro, Brazil; Serviço de Anatomia Patológica/Serviço de Neurocirurgia – Hospital Universitário Clementino Fraga Filho, Universidade Federal do Rio de Janeiro, Rio de Janeiro, Brazil; Universidade Federal do Rio de Janeiro/Macaé, 27930-560 Macaé, Brazil; D’Or Institute for Research and Education (IDOR), Rio de Janeiro, Brazil; National Center of Structural Biology and Bioimaging (CENABIO), 22281-100 Rio de Janeiro, Brazil

**Keywords:** Glioblastoma, Microglia, Angiogenesis, Reactive gliosis

## Abstract

**Background:**

Glioblastoma (GBM) is the most common primary brain tumor and the most aggressive glial tumor. This tumor is highly heterogeneous, angiogenic, and insensitive to radio- and chemotherapy. Here we have investigated the progression of GBM produced by the injection of human GBM cells into the brain parenchyma of immunocompetent mice.

**Methods:**

Xenotransplanted animals were submitted to magnetic resonance imaging (MRI) and histopathological analyses.

**Results:**

Our data show that two weeks after injection, the produced tumor presents histopathological characteristics recommended by World Health Organization for the diagnosis of GBM in humans. The tumor was able to produce reactive gliosis in the adjacent parenchyma, angiogenesis, an intense recruitment of macrophage and microglial cells, and presence of necrosis regions. Besides, MRI showed that tumor mass had enhanced contrast, suggesting a blood–brain barrier disruption.

**Conclusions:**

This study demonstrated that the xenografted tumor in mouse brain parenchyma develops in a very similar manner to those found in patients affected by GBM and can be used to better understand the biology of GBM as well as testing potential therapies.

**Electronic supplementary material:**

The online version of this article (doi:10.1186/1471-2407-14-923) contains supplementary material, which is available to authorized users.

## Background

Glioblastoma (GBM) represents the most common primary brain tumor and the most aggressive glial tumor, leading to poor prognosis for patients in whom the average survival is 12 to 14 months after diagnosis, according to the World Health Organization (WHO). Tumor mass is highly heterogeneous, being composed of several cell types that include not only neoplastic cells, but also normal astrocytes and microglia, as well as cells recruited from the bloodstream such as endothelial cells, monocytes, and lymphocytes [1–3].

Because most GBM symptoms are non-specific, GBM diagnosis may be suggested by MRI exams, but can only be confirmed by histopathological analysis [4–6], which in most cases is done when patients are already in advanced stages of the disease. Contrast enhancement and necrotic/hemorrhagic spots are the main outputs obtained with MRI. In addition, GBM is one of the most angiogenic tumors [7, 8]. The presence of glomeruloid vessels is an important feature for diagnosis through histopathological analysis, as well as cellular atypia, necrosis, and mitotic figures [9, 10].

Glioblastoma progression is highly impacted by the brain microenvironment. Microglia, brain macrophages, and infiltrating macrophages associated with this tumor compose approximately 30% of tumor mass, and display an amoeboid morphology typical of activated macrophagic cells [11, 12]. Astrocytes and endothelial cells also interact with the tumor, triggering key processes such as reactive gliosis and angiogenesis, respectively. Currently, there is a growing body of evidence suggesting that the brain microenvironment as a whole favors GBM growth and spread [2, 13].

At the onset of GBM, microenvironment plays an anti-tumor role; however, once the tumor is established, tumor cells escape immune surveillance and non-cancerous cells begin to play a pro-tumorigenic role [14, 15]. Most current studies on GBM development have been done in nude mice [16], in which the immune response is severely compromised, thus failing to recapitulate some GBM–microenvironment interactions [17]. Thus, an alternative model for studying GBM that takes into account the immune response is much needed for a better understanding of how these interactions take place.

In this study, we developed an orthotopic xenotransplant model of human GBM cells by inoculating immunocompetent mice. Our model presents important features found in GBM patients and may further be used to help develop novel therapeutic strategies to improve the outcome of GBM patients.

## Methods

### Reagents

All culture media components as well as the secondary antibodies conjugated with either Alexa Fluor 488 or Fluor 546 were obtained from Invitrogen–Life Technologies (Carlsbad, CA, USA). All culture plates and flasks were obtained from TPP (Zolstrasse, Trasadingen, Switzerland). Glucose was purchased from Merck (Frankfurter, Darmstadt, Germany), and Fungizone was purchased from Bristol-Meyers Squibb (Princeton, NJ, USA). Rabbit anti- glial fibrillary acidic protein (GFAP) and mouse anti-Vimentin clone V9 antibodies were purchased from DAKO (Produktionsvej, Glostrup, Denmark). Mouse anti-CD31 antibody was purchased from Millipore (Billerica, MA, USA). Biotinylated *Griffonia simplicifolia* Isolectin B4 (IB4) was obtained from Vector (Burlingame, CA, USA), and streptavidin-Cy3 and 4-6-diamino-2-phenylindole (DAPI) were obtained from Sigma (Natik, MA, USA). Mouse anti-IDH1–R132H antibody (clone H09) was purchased from Dianova (Hamburg, Germany).

### Animals

The use of laboratory animals in this study was approved by the Ethics Committee of the Center for Health Sciences (Centro de Ciências da Saúde – CCS) at the Federal University of Rio de Janeiro (Universidade Federal do Rio de Janeiro – UFRJ) (Protocol No. DAHEICB 015). The “Guide for the Care and Use of Laboratory Animals” (published by the National Academy of Science, National Academy Press, Washington, D.C.) was strictly followed in all experiments. All efforts were made to minimize the number of animals used and their suffering. Male Swiss mice (SWR/J) of 10–14 weeks of age, inbred strain were obtained from the Biomedical Sciences Institute at the Federal University of Rio de Janeiro (UFRJ).

### Maintenance of the GBM cell line

The human tumor cell line GBM95 was established in our laboratory [18]. The use of patients’ surgical specimens for the establishment of cell lines for in vitro and in vivo research had the written informed consent from the patients and was approved by the Brazilian Ministry of Health Ethics Committee, under Institutional Review Board (IRB - Research Ethics Committee of Hospital Universitário Clementino Fraga Filho) consent CEP-HUCFF No. 002/01.

Cells were grown and maintained in DMEM-F12 supplemented with 10% FBS. Culture flasks were maintained at 37°C in a humidified 5% CO_2_ and 95% air atmosphere. Cells displaying exponential growth were detached from the culture flasks with 0.25% trypsin/ethylene-diamine tetraacetic acid (EDTA) and seeded. Cultured GBM95 cells were immunoreactive for GFAP, vimetin and nestin [18], but not labeled by IB4 (microglial marker; not shown).

### Maintenance of the human astrocyte cells

Adult primary human astrocytes were isolated from surgically resected anterior temporal lobe tissue, from patients selected for surgical treatment of temporal-lobe epilepsy associated with hippocampus sclerosis. The pathological tissue targeted in surgery for these cases is the gliotic hippocampus, and the anterior temporal lobe resection is used merely as a surgical pathway to the diseased area. All patients gave written consent to the use of their surgical specimens for isolation of cortical cells (including astrocytes) in the study, and the procedures were approved by the Brazilian Ministry of Health Ethics Committee under IRB consent (CEP-HUCFF No. 060/05). As previously described [19], only healthy cortical tissue was used to produce astrocyte cultures. Briefly, tissues were washed in DMEM medium, mechanically dissociated, chopped into small pieces with a sterile scalpel, and incubated in 10 mL of 0.25% trypsin solution at 37°C for 10 min. After centrifugation for 10 min, the cell pellet was resuspended in DMEM/F12 growth medium supplemented with 10% fetal calf serum (FCS), and plated onto tissue culture plates in a humidified 5% CO_2_, 95% air atmosphere at 37°C for 2 hours in order to achieve adherence of microglial cells. The nonadherent astrocytes were transferred into other culture plates, previously coated with poly-L-lysine. Adherent astrocytes were allowed to grow by replacing the medium once a week. New passages of cells were generated by harvesting confluent astrocyte cultures using trypsin-EDTA solution (0.25% trypsin with EDTA; Invitrogen, Carlsbad, CA, USA). Human astrocytes from up to the third passage were used in this study and expressed the human leukocyte antigen (HLA) and typical astrocyte markers, such as GFAP and glutamate-aspartate transporter (GLAST) attesting to their human and astrocytic nature [19].

### In vivo *mouse glioma model*

Male Swiss mice of 10–14 weeks of age weighing 30–35 grams were used. Mice were anesthetized with diazepam (5 mg/kg i.m.), ketamine (100 mg/kg i.m.), and xylasine (25 mg/kg i.m.), and then a brain midline incision was made on the scalp. A small hole was drilled in the skull at stereotaxic coordinates: 1 mm posterior to the bregma and +2 mm mediolateral from the midline. 5 × 10^5^ GBM95 cells (or human astrocytes – control) were delivered in 3 μL DMEM-F12 at a depth of 3 mm with a Hamilton (Hamilton, Reno, Nevada, USA) syringe over 30 minutes. Animals were followed and analyses were done after 14 days after tumor cell injection. Four animals per group (GBM or astrocytes) were used for each experiment described below.

### Magnetic resonance imaging

Magnetic resonance imaging (MRI) was performed 2, 7, and 14 days after tumor cell injection. Mice were anesthetized with ketamine (100 mg/kg i.m.) and xylazine (25 mg/kg i.m.) and images were acquired with a 7-T magnetic resonance scanner (7 T/210 horizontal Varian scanner, Agilent Technologies). Brain images were obtained using a Fast-Spin-Echo (FSE) T2 weighted (TE/TR: 15/2000 ms; matrix: 128×128; slice thickness: 1 mm; with no gap; 12 averages), a FSE proton density (PD) (TE/TR: 10/2000 ms; matrix: 128x128; slice thickness: 1 mm; no gap; 12 averages) and Spin-Echo (SE) T1 weighted (TE/TR: 15/250 ms; matrix: 128x128; slice thickness: 1 mm; with no gap; 12 averages) sequences in the axial (field of view: 21.3 mm × 22.3 mm, in plane resolution: 0,166 mm / 0,174 mm), coronal (field of view: 25.4 mm × 25.4 mm, in plane resolution: 0,198 mm / 0,198 mm), and sagittal (field of view: 25.6 mm × 25.6 mm, in plane resolution: 0,20 mm / 0,20 mm) planes, before and after gadolinium injection (0.05 M/Kg i.p.).

Prior to image analysis, datasets were inspected for artifacts and the brain morphology and tumor characteristics were evaluated. Data processing was performed using MRIcro-Software (http://www.mccauslandcenter.sc.edu) in order to quantify MRI-hyperintensity volume tumor in each animal scanned 7 days and 15 days after human GBM injection. Regions of interest were manually defined on consecutive slices by two investigators on T2-weighted images before gadolinium administration and PD and T1 images after gadolinium injection, obtained from three independent experiments. Graphics were assembled using GraphPad Prism 5.

### Tissue processing

Fourteen days after tumor cell injection, mice were anesthetized and transcardially perfused with 4% paraformaldehyde (PFA) in phosphate-buffered saline (PBS) for perfusion-fixation. Brains were dissected, post-fixed in cold 4% PFA for 24 hours, and stored at 4°C before processing. Tissues were dehydrated in graded ethanol series (30%, 40%, 50%, and 60% for 30 minutes, and then 70%, 80%, and 90% for 1 hour, and finally 100% twice for 1 hour each time), followed by xylene overnight at room temperature. Brains were then embedded in paraffin for 3 hours at 67°C. Coronal sections were cut (5 μm thick) on a microtome. Slices were stained with hematoxylin and eosin and photographed using Nikon Eclipse T300 and LABOMED Luxeo 4D microscopes. The original hematoxylin-eosin stained histopathological slices of the patient’s biopsy upon which the diagnosis of glioblastoma was made were also retrieved from pathology files (Hospital Universitário Clementino Fraga Filho (HUCFF)/UFRJ) and reviewed, as well as photographed using the same microscope.

### Immunohistochemistry

For immunohistochemistry analysis, brains were quickly excised after perfusion-fixation as described above and serially sectioned at 50 μm. The sections were washed with PBS and incubated with 10% NGS diluted in PBS with 0.3% triton X-100 for 90 minutes. They were then incubated with GFAP (1:400), CD31(1:100), Vimentin (1:400) antibodies and with biotinylated IB4 (1:100) overnight at 4°C, then washed again with PBS and incubated with secondary antibodies conjugated with Alexa Fluor 488 or 546 (1:400) and streptavidin-Cy3 (1:400) for 2 hours. The sections were counterstained with DAPI and coverslipped with fluoromount. Negative controls were performed with non-immune rabbit IgG. Slices were imaged using a confocal microscope (Leica TCS-SP5) equipped with a 63x NA 1.40 oil-immersion objective. Image processing was done using Adobe Photoshop. Immunohistochemical staining was also performed with IDH1 antibody (1:10,000) in 4 μm thick tissue sections from paraffin blocks. The Universal LSAB™2 Kit/HRP, rabbit/Mouse-K0675 (Dako, Carpenteria, CA, USA) detection system was used. Negative control consisted of the reaction performed without primary antibody and positive control consisted of a case of grade II oligodendroglioma.

## Results

### Human glioblastoma xenograft growth in immunocompetent mice brain

**Figure 1:**
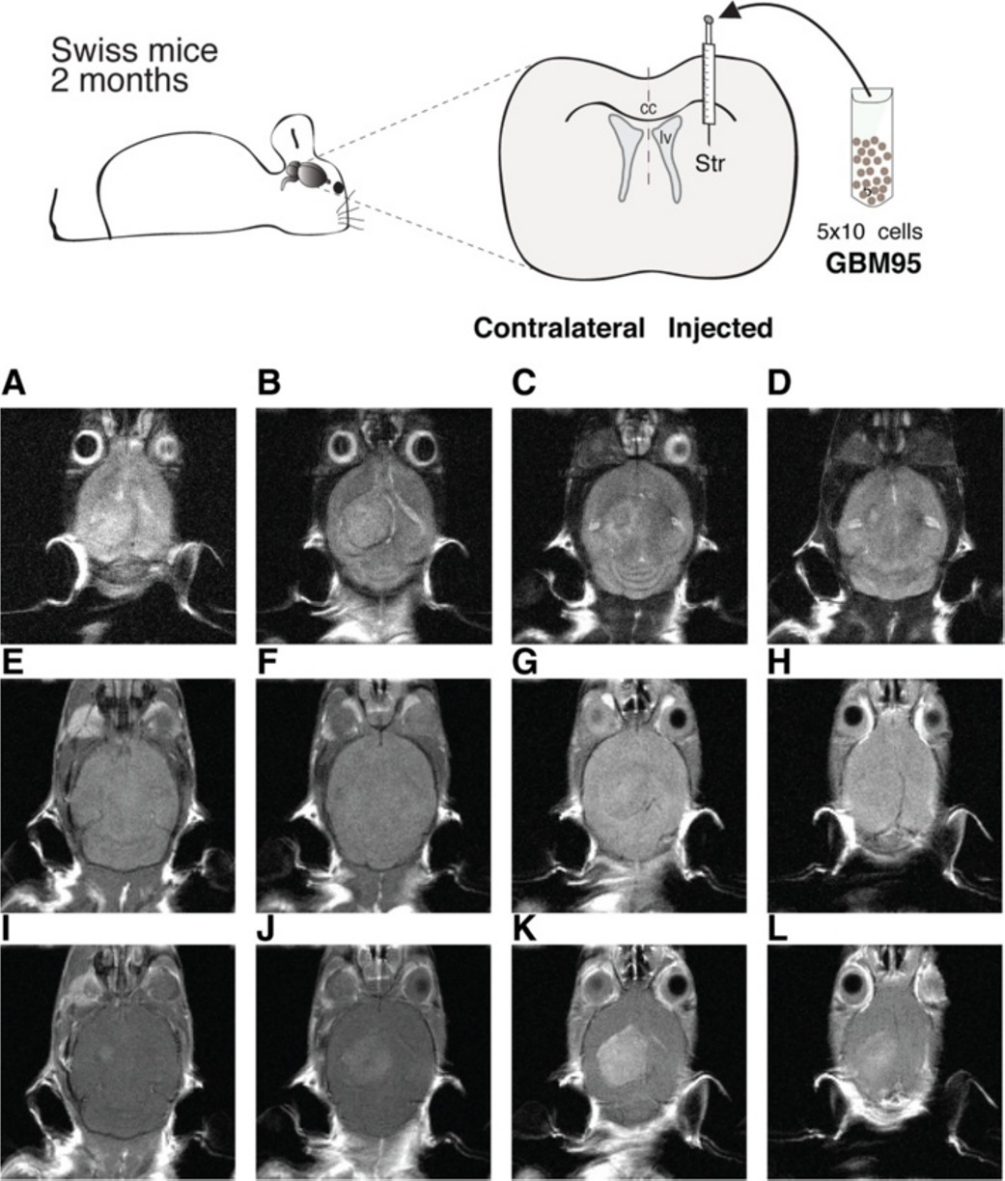
**Magnetic resonance imaging (MRI) 14 days after GBM95 cell transplantation into one representative mouse brain. (A–D)** T2 superior-inferior sequence of the mouse brain shows a shift in brain midline and collapse of lateral ventricles. **(I–L)** T1 superior-inferior sequence after contrast administration, enhanced contrast reflecting blood–brain barrier disruption in comparison to T1 sequence before contrast administration **(E–H)**. Scheme depicts the injection site of GBM95 cells in mouse brain. Data are representative of four separate experiments.

**Figure 2:**
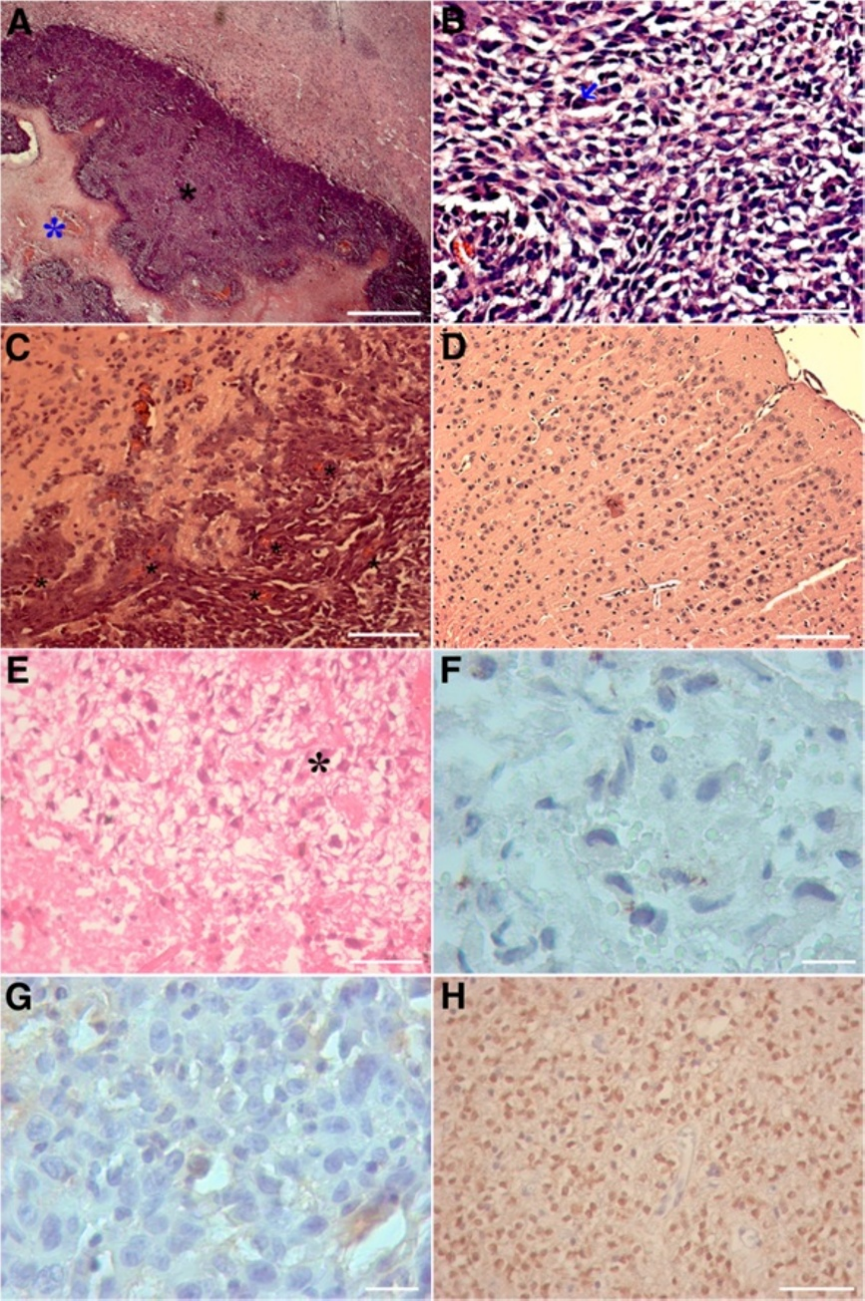
**Histopathological and immunohistochemical characteristics of the tumor mass within mouse brain parenchyma 14 days after cell implantation, and of the original patient’s biopsy. A**, Neoplastic cells forming a circumscribed solid tumor mass into brain tissue (black asterisk); the blue asterisk indicates a necrosis area in the core of the tumor mass. **B**, Neoplastic cells showing prominent anaplasia and mitotic figures (arrow > inset). **C**, Glomeruloid vessels (*). **D**, Human astrocytes inoculated in mouse brain; no tumoral mass is formed. Data are representative of four separate experiments. **E**, Microscopic analysis of the patient’s original biopsy showing anaplastic cells (*, top right) and tumoral necrosis (bottom left). **F** and **G**, Negative immunostaining for IDH1–R132H mutation in both tumor mass within mouse brain parenchyma **(F)** and in patient’s biopsy material **(G)**. **H**, Grade II oligodendroglioma, a positive control case of the IDH1–R132H mutation. Scale bars, 100 μm **(A)**; 50 μm **(B, E, H)**; 80 μm **(C,** **D)**; 30 μm **(F,** **G)**.

In order to evaluate human GBM progression in immunocompetent mouse brains, we performed MRI and histopathological analysis. MRI performed 2 days after GBM cell implantation did not reveal blood–brain barrier (BBB) disruption (data not shown). However, MRI performed 7 and 14 days after tumor cell injection confirmed tumor growth and mass formation with BBB disruption (Figure 1J–L; Additional file 1). Figure 1 shows an axial view of a tumor-bearing brain and Additional file 1 shows the increase of the tumor mass volume at 7 and 14 days after cell injection. In addition, two weeks after GBM cell injection, the MRI also revealed hemorrhage and necrosis in the core of the tumor mass, which was confirmed by later histological analyses. These MRI aspects are similar to those commonly found in patients with GBM [5]. Furthermore, histological analyses showed that the xenografted tumor infiltrates the brain parenchyma, forming a solid tumor mass (Figure 2A), and presents all microscopic histopathological features required for the diagnosis of GBM according to the WHO classification [1, 4, 20], namely cellular atypia, presence of mitotic figures, endothelial vascular proliferation including formation of glomeruloid vessels, and/or necrosis (Figures 2A–C). The same characteristics were detected in the patient’s original biopsy material (Figure 2E). In contrast, injections of healthy human astrocytes did not induce tumor mass development at 14 (Figure 2D) or 30 days (see Additional file 2) after injection of these cells. In addition, we performed xenografts using another human tumor cell line, the GBM02 [18] injected in a distinct mouse strain (C57/Black6), and the results were similar to those found in this work (data not shown).

### Xenografted GBM cells show the same negative result for IDH1–R132H mutation as the original tumor

Since glioblastoma cases negative for IDH1 mutation tend to be those primary and more aggressive glioblastomas [21], we evaluated the presence of IDH1–R132H mutation by immunohistochemistry in order to verify if the tumor cells injected in mouse brain tissue were able to maintain this same characteristic from the original tumor. Both materials proved to be negative for IDH1–R132H mutation (Figure 2F–H).

### Xenografted GBM cells express human vimentin and promote reactive gliosis in mouse brain tissue

**Figure 3:**
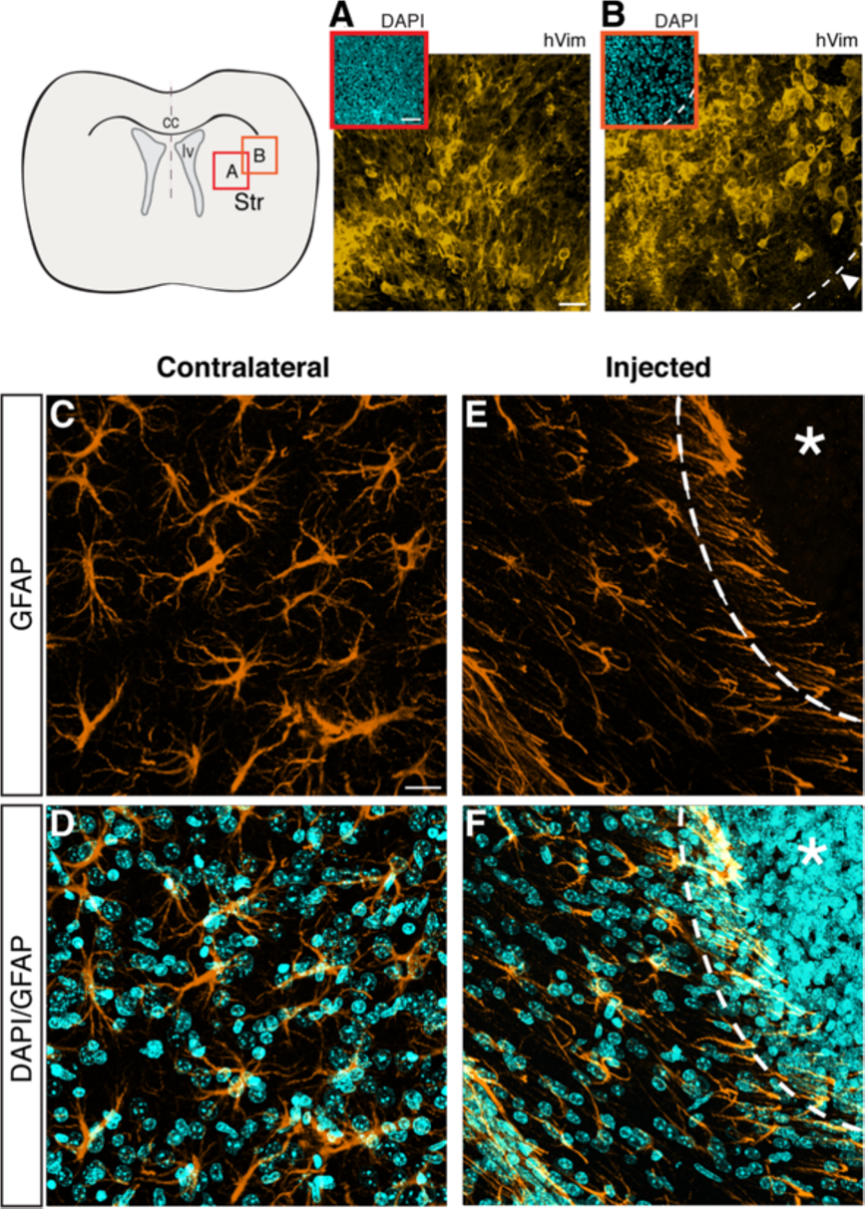
**The tumor expresses human vimentin (hVim) and induces reactive gliosis in the adjacent brain parenchyma.** GBM95 cells were injected in the striatum of immunecompetent mice 14 days before the immunohistochemical analysis. hVim staining (orange) at the core of the tumor mass **(A)**, depicted by cell nuclei atypia (DAPI counterstaining in cyan, inset) and at the border of the tumor mass (**B**, delimited by dashed line, inset, and arrowheads) attest human origin of the tumor. **(C–D)** GFAP immunoreactivity cells in the contralateral hemisphere exhibit a stellate morphology. **(E–F)** In contrast, in the injected hemisphere, GFAP^+^ cells display a palisade arrangement of cell processes, which irradiate from the core of the tumor mass (delimited by the dashed line and indicated by the asterisk). Data represent four separate experiments. Scale bar, 40 μm. cc = corpus callosum; lv = lateral ventricle; Str = striatum.

We confirmed the human origin of the tumor in the mouse brain two weeks after inoculation using a specific antibody that recognizes human vimentin (Figure 3A, B). As expected, no vimentin^+^ cells were observed in the contralateral hemisphere (not shown). GBM induces reactive gliosis in surrounding brain tissues, which is characterized by morphological changes, increase in GFAP immunoreactivity and cellular distribution, besides the release of pro-inflammatory cytokines [22, 23]. In Figure 3E–F, GFAP^+^ reactive cells present an unusual palisade-like distribution irradiating from the borders of tumor mass, whereas GFAP-stained astrocytes present regular morphology in the contralateral hemisphere (Figure 3C, D).

### Xenografted tumor is highly angiogenic

**Figure 4:**
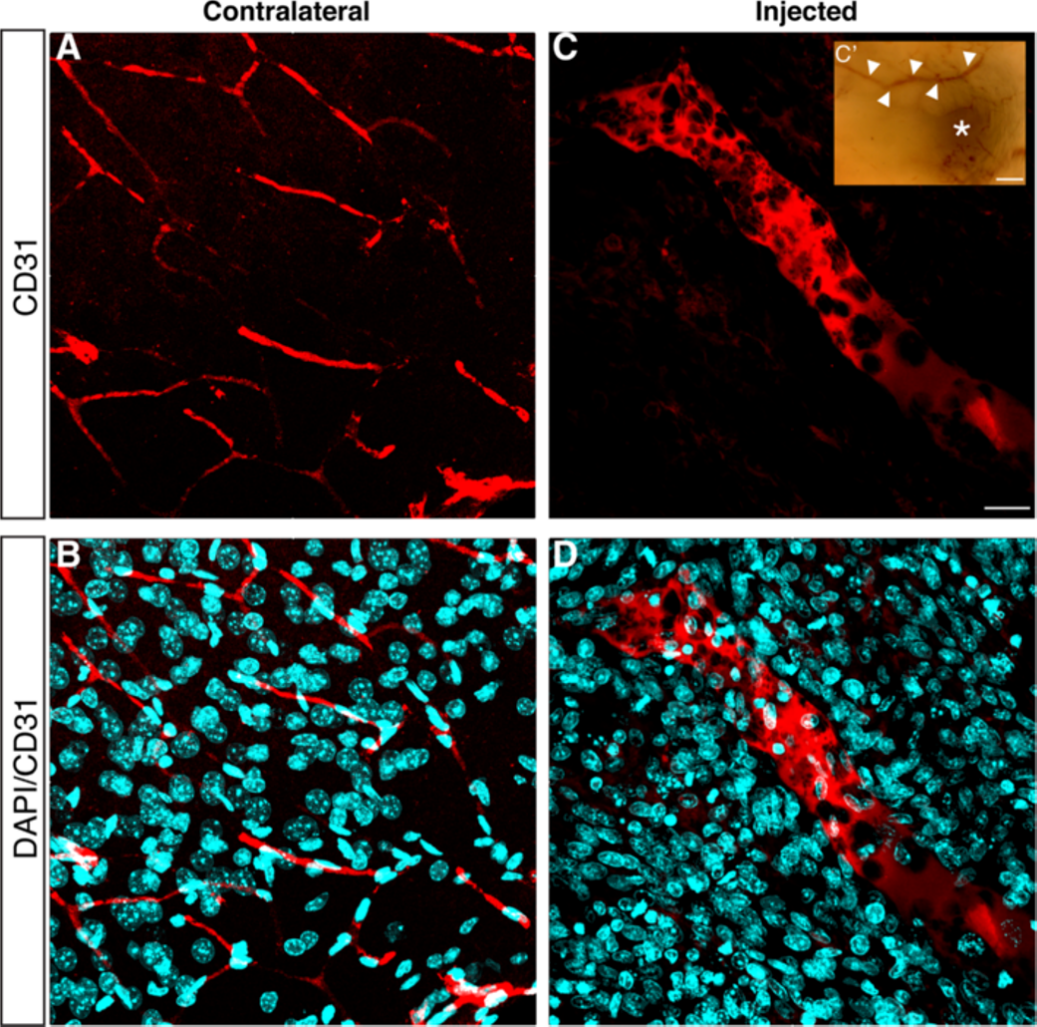
**Human GBM is highly angiogenic. (A** **–** **B)** CD31 immunostaining (red) shows blood vessels displaying a regular morphology in the contralateral hemisphere. **(C** **–** **D)** Disrupted wall of an irregular blood vessel (CD31^+^) in the injected hemisphere demonstrates a chaotic angiogenesis. Cell nuclei counterstaining by DAPI (cyan). (C’) Macroscopic view of freshly dissected brain reveals the presence of irregular blood vessels (arrowheads) and necrotic area (asterisk). Data represent four separate experiments. Scale bar, 40 μm.cc = corpus callosum; lv = lateral ventricle; Str = striatum.

Glioblastoma is one of the most angiogenic tumors, although a marked imbalance between pro- and anti-angiogenic factors in the tumor microenvironment results in aberrant vessels [24]. We observed a high expression of CD31 associated with abnormal blood vessels with fenestrated walls and variable diameter. The variable diameter can also be noticed in the dissected tissue, indicating profuse angiogenesis (Figure 4). Near the necrotic area, we can visualize enucleated endothelial cells along large caliber blood vessels (Figure 4C, D). CD31 expression reveals regular blood vessels in the contralateral hemisphere (Figure 4A, B).

### Microglia are recruited to human GBM site

**Figure 5:**
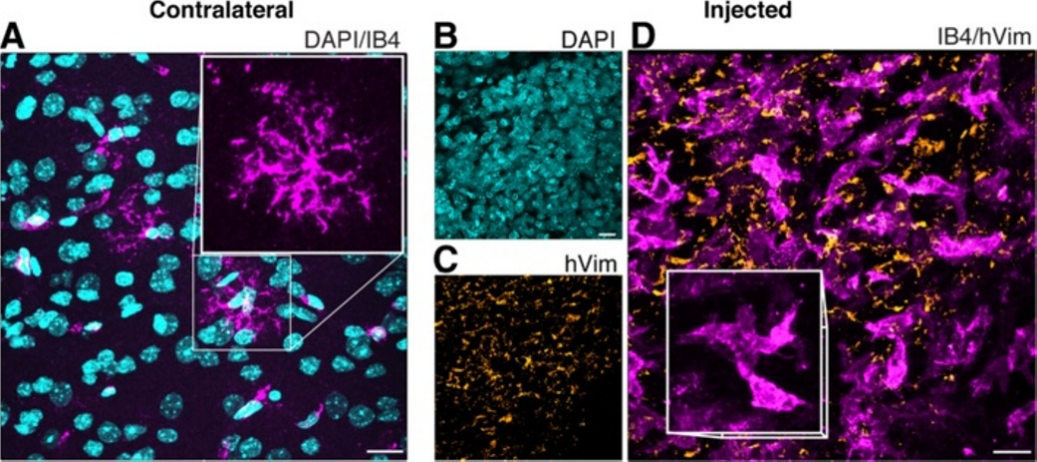
**Microglial cells are recruited from the mouse brain parenchyma. A**, in the contralateral hemisphere we noted ramified microglia (IB4, magenta; DAPI in cyan), whereas in the core of the tumor mass (**B**, DAPI in cyan) we observed hVim^+^ tumor cells (**C**, orange). **D**, In contact with GBM (hVim^+^ cells, orange), infiltrated microglia exhibit amoeboid morphology (IB4, magenta). GBM95 cells were injected in the striatum of immunecompetent mice 14 days before the immunohistochemical analysis. Data represent four separate experiments. Scale bars, 40 μm. hVim = human vimentin. Cc = corpus callosum; LV = lateral ventricle; Str = striatum.

In contact with tumor cells, microglia infiltrate tumor mass and acquire an amoeboid phenotype typical of activated macrophages [25], as seen in Figure 5D, revealed by IB4 staining. Notice that positive-vimentin (tumor) cells are not labeled for IB4 (Figure 5C), indicating that recruited microglia are exclusively originated from brain parenchyma. As shown in Figure 5A, contralateral hemisphere presents ramified cells, characteristic of resident microglia.

## Discussion

In this study, we used an ortothopic xenotransplant model in immunocompetent mice that was able to recapitulate the human GBM features as described by the WHO [4, 26].

Current studies show that using orthotopic allotransplants of murine glioma cell lines in immunocompetent animals results in a prominent tumor mass [27, 28]. Nevertheless, the histopathological features present in these tumors do not reproduce the ones described in GBM patients, suggesting that the allotransplanted tumor in mice cannot be compared to an authentic human GBM [29].

In our model, despite the incompatibility of major histocompatibility complex (MHC), we observed the development of a tumor mass in immunocompetent mice inoculated with human GBM cells. MRI analysis of xenografted mice showed a growing tumor mass (Additional file 1) which enhanced with MR contrast, evidencing, BBB disruption (Figure 1), and the presence of necrotic/hemorrhagic spots in the core of the tumor mass, similar to what is described in GBM patients [10]. Moreover, we also observed the same histopathological features present in GBM patients [9] as described by the WHO (Figure 2). These similarities are compatible with the development of human GBM tumor, as also indicated by the presence of human vimentin-positive cells in the entire tumor mass (Figure 3A).

Interestingly, although the present model shares several imaging and histological features with the GBM commonly found in patients, we noticed that the borders of tumor mass in the mouse brain were rather circumscribed and not infiltrative (Figure 2A) like those generally found in GBM patients. This difference, observed by us and other groups that used in vivo glioma models [30–32], may be due to a mismatch in cell surface recognition proteins, mainly MHC, between mice and humans [33]. Nevertheless, these probable differences in the tumor border in the mice did not affect tumor development, which matched the diagnosis criteria for GBM. Moreover, the fact that the tumor has well-defined border (Figure 2A) does not invalidate the diagnosis of a malignant neoplasia. Indeed, it resembles the so-called malignant glioneuronal tumor (MGNT) described by the team of Dr. Daumas-Duport as a more superficial and fairly well-defined tumor, although highly aggressive, causing recurrence and patient death [34]. The MGNT is classified by the WHO as a GBM.

The humanized mouse model, in which immunodeficient mice are engrafted with human hematopoietic cells or tissues, or mice that transgenically express human genes [35, 36], could be an alternative to deal with the problem of the mismatch between mouse and human MHC. Despite the advantages of this model, humanized mice are onerous and they still present biological constraints that could impair the proper function of its immune response, i. e., innate immunity defects such as decrease in macrophage function [37] and the lack of human-specific adhesion molecules to improve appropriate traffic of human cells [38].

We also verified that both the patient’s biopsy material as well as the xenografted GBM cells injected into mouse brain were negative for IDH1–R132H mutation. This recently described mutation in isocitrate desidrogenase enzyme type 1 (IDH1) seems to be frequent in diffuse gliomas of astrocytic and oligodendroglial lineage, as well in those secondary glioblastomas that derive from such tumors [39]. In contrast, those primary and more aggressive glioblastomas are generally negative for IDH1 mutation, [21] and it seems that IDH-mutation is related to prognosis. Our results are in accordance with those already described, since this was a case of primary glioblastoma, and also show that xenografts are able to keep the IDH1 mutation status of highly aggressive tumors. Although there are other types of IDH1 mutation, the antibody used detects the most common IDH1 mutation, which occurs in approximately 90% of cases, the R132H [40]. Thus, not only are xenotransplant cells able to reproduce histopathological characteristics of malignancy found in glioblastomas, they can also reproduce the molecular status of one the most important and recently described molecular markers of prognosis in glioblastomas.

Reactive gliosis is triggered by brain injuries and mainly consists of morphological changes and increase in GFAP immunoreactivity [22, 23]. As expected, we observed GFAP^+^ cells displaying a palisade-like arrangement in contrast to stellate astrocytes, which were distributed in the contralateral hemisphere and not in the tumor area (Figure 3A). We also observed that our ortothopic xenotransplant model produced a highly angiogenic tumor mass, which is known to be essential to deliver nutrients and oxygen to the tumor [24, 41]. Additionally, we observed defective CD31^+^ vessels that presented fenestrated walls and variable calibers (Figure 4), indicating that angiogenesis in the tumor mass is aberrant, as described in GBM patients [8, 42]. Altogether, these results indicate that this is a promising animal model for the study of human GBM progression in vivo.

GBM triggers BBB disruption leading to the invasion of circulating monocytes that ultimately differentiate into macrophages. These macrophages and locally recruited microglia integrate into the tumor mass and are known as glioma-associated microglia/macrophages (GAM) [43, 44]. GAM play pivotal roles in GBM development, affecting glioma growth, spread, angiogenesis, and local immunosuppression [25, 30]. In our study we observed a massive infiltration of IB4^+^ cells displaying amoeboid phenotype, characteristic of activated macrophagic cells (microglia and macrophages) (Figure 5). Although IB4 is not a specific macrophage marker because it recognizes glycan moieties that are also present in the endothelial cell surface, we did not observe blood vessels labeled by IB4 in the tumor mass, possibly due to the fact that vessels within the tumor are not exclusively derived from endothelial cells, but also from tumor cells [7, 8].

Xenotransplant GBM models are currently performed mostly in nude mice in which inoculated cells are able to originate a tumor mass that nevertheless fails to reproduce all the tumor stroma. Additionally, the tumor may not present the main histopathological hallmarks of GBM [45, 46]. Although nude animals are widely used, they represent a limited model to investigate the interactions established between immune cells, particularly recruited monocytes, during tumor progression [17, 47]. In contrast, our orthotopic xenotransplanted model allows fully comprehensive studies on the interactions established between tumor cells and GAM. For instance, it may allow unveiling potential events triggered by immune responses and aimed at preventing tumor formation. Additionally, our model may also be used to investigate the hypothesis that cellular interactions and the release of soluble inflammatory mediators in the tumor microenvironment are coopted by tumor cells, resulting in GBM progression. These studies would not be possible with nude animals [17, 29].

Furthermore, nude animals are highly vulnerable to the side effects of therapeutic cancer treatments thus hampering their application in pharmacological studies [48–50]. Tests with anti-tumor drugs using our model could have a better outcome than that obtained with nude animals. In fact, we have recently demonstrated that Equinatoxin II, a pore-forming toxin from sea anemones, potentiates the effects of Etoposide in the induction of GBM cell death [51]. In this study, we used human GBM cells xenografted in the striatum of immunocompetent mice.

## Conclusions

Here we report, for the first time, the occurrence of several hallmark features of typical human GBM in a xenotransplant inoculated mouse model. Our model allows the study of molecular and cellular interactions during GBM tumor progression that take place with active immune response and may further be used to help develop novel therapeutic strategies to improve the outcome of GBM patients.

## Electronic supplementary material

Additional file 1: Figure S1: Magnetic resonance image analysis of tumor volume at 7 and 14 days after human glioblastoma xenograft in Swiss mice, showing the dynamics of glioblastoma’s growth. Tumor volumes were measured on T2-weighted (T2) (before gadolinium (Gd) injection) and on proton density (PD) images (after Gd injection). Values are represented by median and standard error. Two-way analysis of variance was used to compare tumor volumes at 7 and 14 days (*p < 0.001). Data are representative of three separate experiments. (PDF 82 KB)

Additional file 2: Figure S2: Injections of human astrocytes did not induce tumor mass development at 30 days after injection of these cells. Hematoxilin–eosin staining of brain tissue. Data represent four separate experiments. Scale bars, 100 μm (A, B); 50 μm (C, D). (PDF 165 KB)

## Abbreviations

GBM: Glioblastoma
MRI: Magnetic resonance imaging
WHO: World Health Organization
IB4: Biotinylated *Griffonia simplicifolia* Isolectin B4
DAPI: 4-6-diamino-2-phenylindole
Universidade Federal do Rio de Janeiro – UFRJ: Federal University of Rio de Janeiro
EDTA: Ethylene-diamine tetraacetic acid
FCS: Fetal calf serum
PFA: Paraformaldehyde
PBS: Phosphate-buffered saline
GFAP: Glial fibrillary acidic protein
MHC: Major histocompatibility complex
BBB: Blood–brain barrier
GAM: Glioma-associated microglia/macrophages.
