# Supplementary figures and images for: The orthotopic xenotransplant of human glioblastoma successfully recapitulates glioblastoma-microenvironment interactions in a non-immunosuppressed mouse model
Source: BMC Cancer. 2014 Dec 8;14:923. doi: 10.1186/1471-2407-14-923 (PMC4295410; doi:10.1186/1471-2407-14-923)

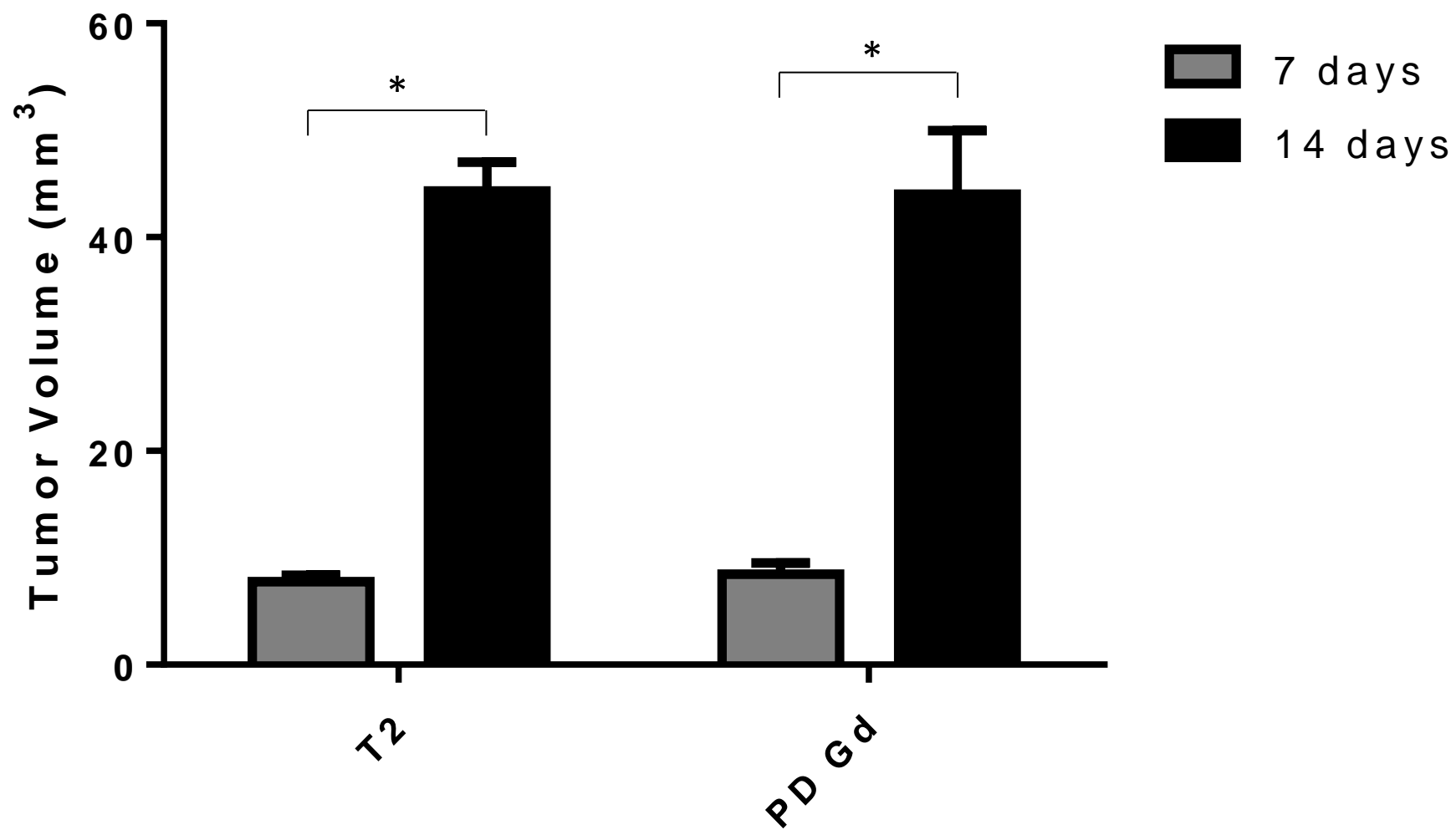

Supplement: Supplementary file 1 — Additional file 1: Figure S1: Magnetic resonance image analysis of tumor volume at 7 and 14 days after human glioblastoma xenograft in Swiss mice, showing the dynamics of glioblastoma’s growth. Tumor volumes were measured on T2-weighted (T2) (before gadolinium (Gd) injection) and on proton density (PD) images (after Gd injection). Values are represented by median and standard error. Two-way analysis of variance was used to compare tumor volumes at 7 and 14 days (*p < 0.001). Data are representative of three separate experiments. (PDF 82 KB) [file 12885_2014_5082_MOESM1_ESM.pdf]

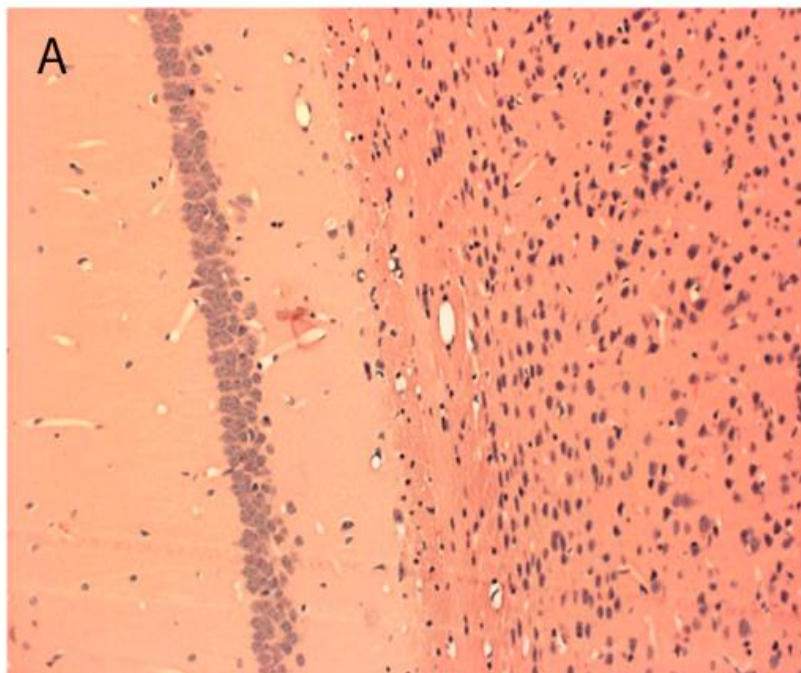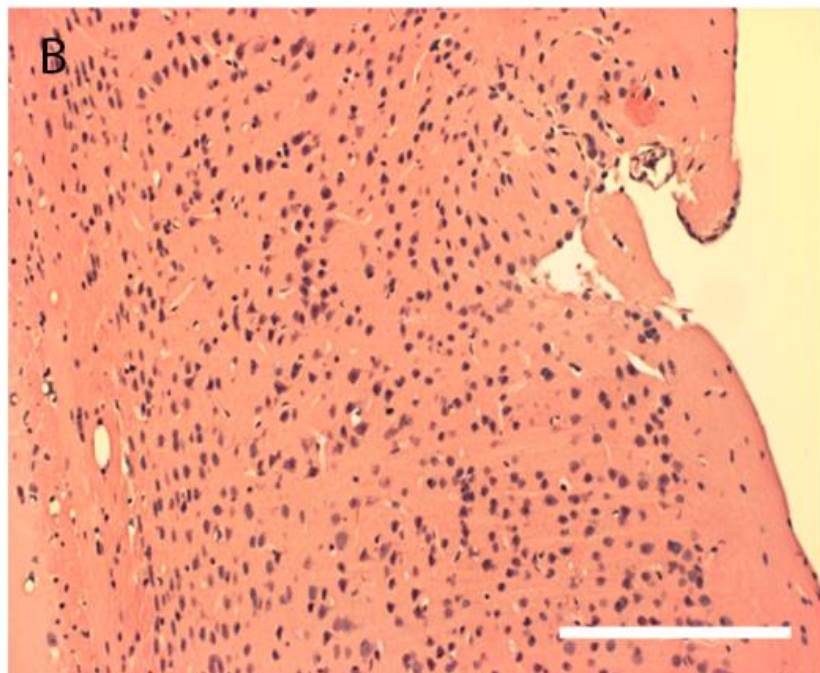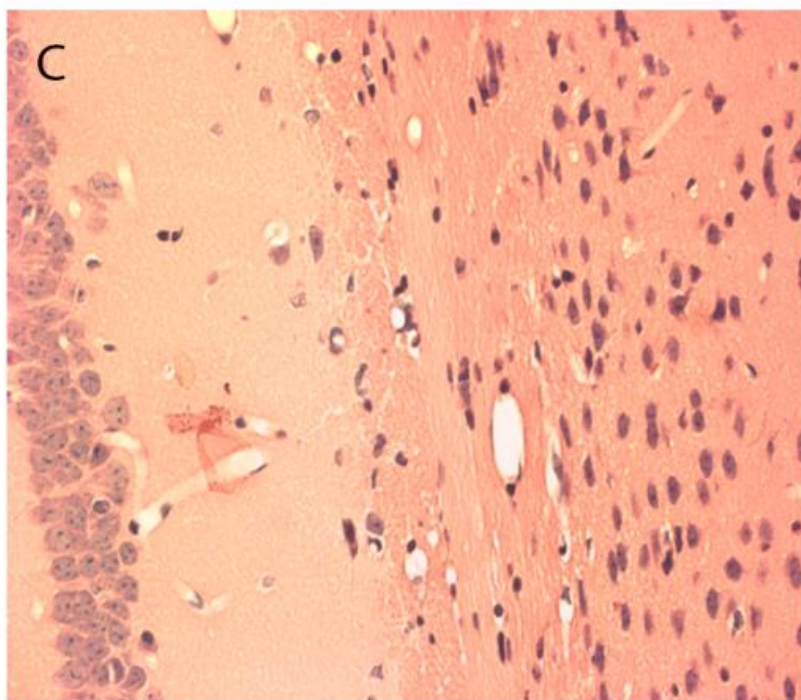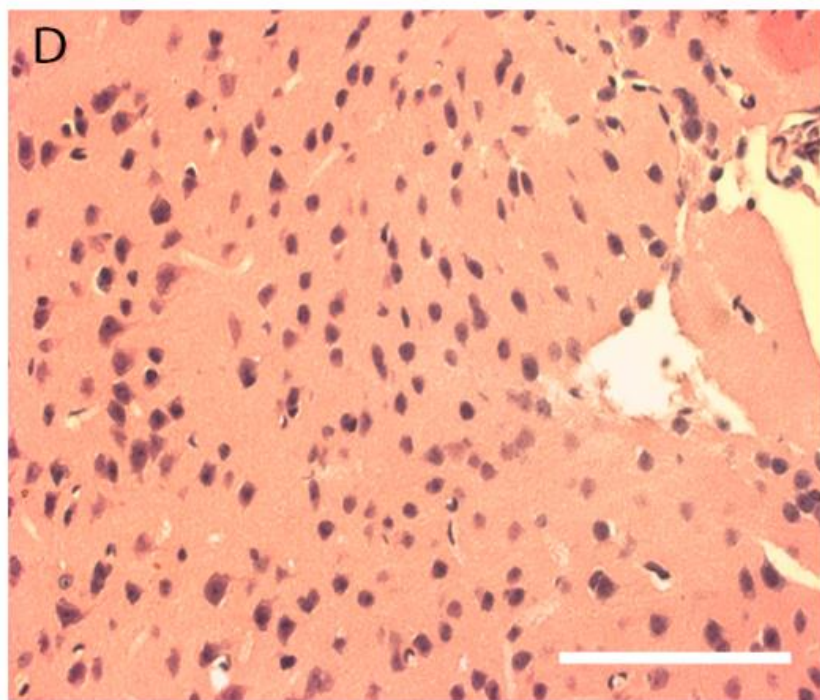

Supplement: Supplementary file 2 — Additional file 2: Figure S2: Injections of human astrocytes did not induce tumor mass development at 30 days after injection of these cells. Hematoxilin–eosin staining of brain tissue. Data represent four separate experiments. Scale bars, 100 μm (A, B); 50 μm (C, D). (PDF 165 KB) [file 12885_2014_5082_MOESM2_ESM.pdf]
